# Unconsciousness from Constipation

**Published:** 1897-04

**Authors:** 


### UNCONSCIOUSNESS FROM CONSTIPATION.

Very stout women have suddenly become unconscious from a long-continued constipation. A physician relates a case in which unconsciousness, with stertorous breathing in the night, simulated an apoplectic attack. By the aid of mustard to the feet and abdomen, with ice to the head, and a large enema of soap, water and castor oil, a large evacuation was procured, with speedy return to consciousness. The slow poison from the retained fecal matter probably brought on unconsciousness.—*Health Magazine.*

